# Global progress in tobacco control: the question of policy compliance

**DOI:** 10.1080/16549716.2020.1844977

**Published:** 2020-11-16

**Authors:** Carrie L. Anderson, Ute Mons, Volker Winkler

**Affiliations:** a Institute of Global Health, Epidemiology, University of Heidelberg, Heidelberg, Germany; b Cancer Prevention Unit & WHO Collaborating Centre for Tobacco Control, German Cancer Research Center (DKFZ), Heidelberg, Germany; c Faculty of Medicine and University Hospital Cologne, Heart Center, University of Cologne, Cologne, Germany

**Keywords:** Tobacco control policy, compliance, MPOWER

## Abstract

**Background**: Currently, about 65% of the world’s population is covered by at least one MPOWER tobacco control policy measure. The impact of such policies might rely on policy compliance.

**Objective**: This study aims to describe and compare global trends in legislation and compliance of the following three tobacco control policies between 2009 and 2019: direct advertisement, promotion and sponsorship, and smoke-free environments.

**Method**: Data from the six most recent WHO Tobacco Control (2009–2019) reports were used to show the development of and possible associations between legislated policies and policy compliance. Data pertaining to the three indicators direct advertisement, promotion and sponsorship, and smoke-free environments were collected and analysed per country income category, according to the Organization for Economic Co-operation and Development. For each country, we (i) calculated the legislation describing the situation according to the law as a percentage of fulfilled MPOWER measurements and (ii) present the level of compliance (ranging from 0 to 10) for the corresponding policy.

**Results**: Both tobacco control policy legislation and compliance for direct advertising improved worldwide – between 2009 and 2019 the median increased from 37.5% to 87.5% for policy and from 5 to 8 for compliance. In contrast, promotion and sponsorship restrictions hardly developed since 2011 and are especially weak among low- and middle-income countries. With respect to smoke-free environments, global policy legislation increased steadily over time while the relative compliance hardly increased. In 2019 data did not show significant correlations between policy legislation and compliance: direct advertising ρ = −0.003, p = 0.970; promotion and sponsorship ρ = 0.140, p = 0.107; smoke-free environments ρ = 0.158, p = 0.070.

**Conclusion**: There is a clear need to understand the barriers in achieving tobacco control policy compliance and to routinely collect and incorporate data on compliance in research in order to generate a more reliable basis for further improvements in tobacco control.

## Background

There is no doubt about the success and importance of the WHO Framework Convention on Tobacco Control (FCTC) as the first global health public treaty [1]. The convention entered into force in 2005 in order to respond to the globalization of the tobacco epidemic protecting people from the devastating health, social, environmental and economic consequences of tobacco consumption, and secondhand smoke exposure [2]. The MPOWER policy package was introduced in 2008 and consists of the following six evidence-based policy components to help countries implement the FCTC: monitor tobacco use and prevention policies, protect people from tobacco smoke, offer to help quit tobacco use, warn about the dangers of tobacco, enforce bans on tobacco advertising, promotion and sponsorship, and raise taxes on tobacco [3]. Previous research has shown an association between achieved recommended tobacco control policy based on MPOWER measures and a decrease in smoking prevalence [4,5]. The seventh and latest WHO Report on the Global Tobacco Epidemic in 2019 aims to track the status of the tobacco epidemic and interventions to combat it. The report notes that on a global scale, an estimated 5 billion people are covered by at least one MPOWER measure [6]. Although this advancement in policy adoption is certainly a noteworthy achievement, ample room for improvement remains. Scaling up these aforementioned policies and strengthening interventions which are known to work could truly help tobacco users quit [7]. Additionally, what is seldom discussed is how well tobacco control policies are, in fact, adhered to. Often these policies are only described in terms of passed laws, but the true degree of policy implementation may heavily rely on policy compliance. This study aims to describe and compare global trends in legislation and compliance of the following three tobacco control policies between 2009 and 2019: direct advertisement, promotion and sponsorship, and smoke-free environments.

## Methods

Data pertaining to country national legislated policies as well as for the level of policy compliance were collected from the six latest WHO Tobacco Control reports (the first WHO report in 2008 was excluded from this analysis due to the lack of compliance data in the American and European regions). Data were extracted for the following three MPOWER measures: direct advertisement, promotion and sponsorship, and smoke-free environments [6,8]. Country-level achievements in banning tobacco direct advertising were assessed based on whether the bans covered the following types of advertising: national television and radios, local magazines and newspapers, billboards and outdoor advertising, and point of sale (indoor); and promotion and sponsorship bans included free distribution of tobacco products in the mail or through other means, promotional discounts, non-tobacco goods and services identified with tobacco brand names (brand stretching), brand names of non-tobacco products used for tobacco products (brand sharing), appearance of tobacco brands (product placement) or tobacco products in television and/or films, sponsorship (contributions and/or publicity of contributions). The smoke-free data used were based on national legislation when available, and legislation in subnational jurisdictions where available and where national laws are incomplete. Legislation was assessed to determine whether smoke-free laws provided for a complete indoor smoke-free environment at all times, in all the facilities of each of the following eight places: health-care facilities, educational facilities other than universities, governmental facilities, indoor offices and workplaces not considered in any other category, restaurants or facilities that serve mostly food, cafes, pubs, and bars or facilities that serve mostly beverages, and public transport [9]. For each country, we (i) calculated the legislation describing the situation according to the law, and (ii) present the level of compliance for the corresponding policy. In brief, we expressed legislation as a percentage of fulfilled MPOWER measurements by tabulating the percentage of bans on direct advertising, promotion and sponsorship, and the number of smoke-free environments in place per each country. For example, in the 2019 report Algeria had reported bans on smoking in health-care facilities, educational facilities except universities, universities, and in pubs/bars (4 categories), but they did not have reported smoking bans in government facilities, indoor offices, restaurants, public transit, and in all other indoor public places (5 categories). Hence, Algeria was assigned a score of 4/9 = 44% for this year and legislation. Further details regarding the methodology used pertaining to legislated policy calculation can view in a previous research article by Anderson et al. [4]. Compliance data were also collected from the WHO Tobacco Control Reports. As described in the 2019 Technical Note I, compliance scores were assigned by up to five national experts per country, who independently scored the compliance as ‘minimal’, ‘moderate’, or ‘high’. Experts were selected based upon the following criteria: person in charge of tobacco prevention in the country’s ministry of health or the most senior government official in charge of tobacco control of tobacco-related conditions; the head of a prominent non-governmental organization for moderately enforced policies and no points for minimally enforced policies, with a potential minimum of 0 and maximum of 10 points in total from these five experts [9]. Countries with missing data on compliance were excluded from the analysis of the corresponding policy, in order to maintain the best coherence possible. Further, we grouped countries according to the following four income categories defined by the Organization for Economic Co-operation and Development (OECD): Least-Developed Countries (LDC), Low- and Middle-Income Countries (LMIC), Upper- and Middle-Income Countries (UMIC), and High-Income Countries (HIC) [10]. Data were presented graphically to depict the progression of each indicator throughout the years of the Tobacco Control report. The median scores per income category and an overall global median were indicated on the graph by lines. In a second Figure, we showed the relation of 2019 policies with compliance for each of the three indicators also displaying Spearman’s correlation coefficients and the linear association. Correlation analyses were conducted to explore associations between compliance and legislation per policy domain.

## Results

**Figure 1.**
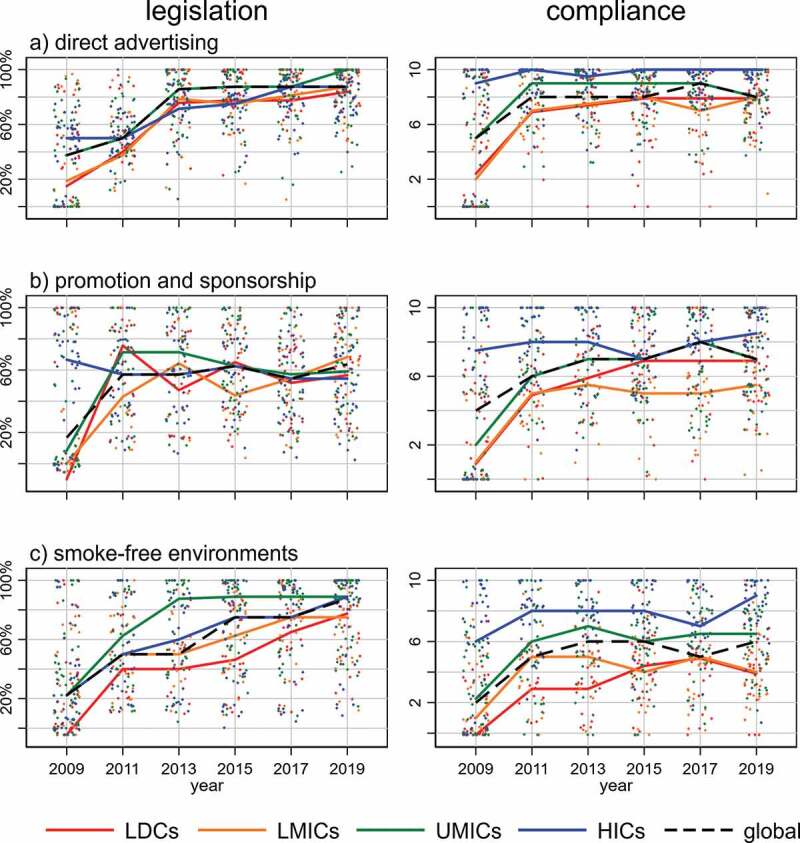
Percentage of legislated policies and level of compliance globally and by income categories, each dot represents a country and medians are indicated by lines. Year refers to the year in which data was acquired (i.e. data from the 2019 report was collected in 2018)

Figure 1 presents data pertaining to legislated policies and to policy compliance while the colouring indicates OECD income categories. Overall, results indicate a positive development for the three MPOWER measures with regards to both legislation and compliance. Although particular increases were seen in all indicators post-2009, observing the data with enhanced scrutiny raises some concerns. While legislation for direct advertising and smoke-free environments strongly improved and reached a median of 88% globally in 2019, compliance developed differently. For direct advertising, global compliance reached a high median score of 8 and even 10 among HICs, whereas the median global value for smoke-free environments lingered at 6. Looking separately by income group, median smoke-free compliance in both LDCs and LMICs remained at 4. The MPOWER measure promotion and sponsorship show a similar development with respect to legislation and compliance, hardly improving since 2011. LMICs are performing worst with respect to compliance for this measure.

**Figure 2.**
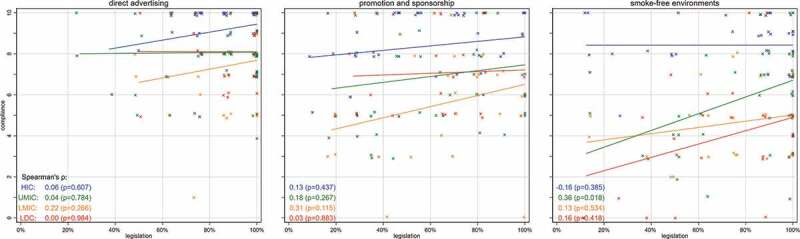
Correlation of legislated policies with level of compliance by income categories, each dot represents a country, medians are indicated by lines, data from 2019 WHO report on the global tobacco epidemic

Figure 2 depicts the correlations between the 2019 legislated policies with their corresponding compliance levels for direct advertising, promotion and sponsorship, and smoke-free environments, by OECD income category. A low correlation was observed between legislation and compliance for all three indicators: direct advertising ρ = −0.003, p = 0.970; promotion and sponsorship ρ = 0.140, p = 0.107; smoke-free environments ρ = 0.158, p = 0.070. Income category-specific correlations did not reveal significant associations either.

## Discussion

Even though data on compliance is limited (for 2019 compliance data are missing for about 30% of the countries), our analysis reveals major differences between legislation and compliance, especially in LDCs and LMICs. The less than ideal level of overall compliance with tobacco control policies could partially be due to weak national enforcement. Additionally, the design of the compliance monitoring system itself is subjective, and thus possibly susceptible to both subjectivity and social desirability biases as national experts are responsible for providing such compliance rankings. In order to identify avenues to improve compliance with tobacco control laws, it is imperative to understand the reasoning behind weak rankings. Although only limited research has been conducted, some studies investigated factors associated with achieving high compliance of national smoke-free laws. For example, compliance with national smoke-free laws was positively associated with government involvement (training and guidelines for inspections) and perceived corruption control [11].

Recommendations on strategies to improve enforcement of smoke-free laws and tobacco advertising, promotion and sponsorship bans can be found in the FCTC guidelines on Articles 8 and 13, respectively [12,13]. These mainly include setting up an enforcement infrastructure (such as designating an independent authority as well as monitoring and reporting systems), effective sanctioning mechanisms, and mobilisation of the public and civil society to report violations. However, such strategies might be especially difficult to implement in LDCs and LMICs, if required resources and capacities are lacking. Dedicated funding and technical support could support such countries in further improving enforcement and compliance.

In conclusion, we propose further research efforts to understand the barriers in achieving policy compliance as well as routine evaluation of policy compliance in addition to tobacco control policy implementation. Compliance ratings could be improved by incorporating active surveillance systems. Efforts could be made to help improve compliance to smoke-free tobacco control policies within UMICs, LMICs, and LDCs. More accurate assessment of the impact of tobacco control policies, and thus a more reliable basis for further improvements in tobacco control is a key to effectively protect people against the adverse health effects of tobacco smoking.

## Paper context

Although research has shown that global tobacco control policy legislation based on the WHO Framework Convention on tobacco control is improving since the initial FCTC implementation, the degree of global policy compliance is poorly understood. Here we showed less than ideal compliance to tobacco control legislation over time in least developed, low- and middle-income, upper- and middle-income and high-income countries. Research efforts to understand the barriers in achieving policy compliance and further evaluation of policy compliance are recommended.
